# Effects of Supplemental Drugs on Hexaminolevulinate (HAL)-Induced PpIX Fluorescence in Bladder Cancer Cell Suspensions

**DOI:** 10.3390/ijms23147631

**Published:** 2022-07-10

**Authors:** Kit Man Chan, Krasimir Vasilev, Melanie MacGregor

**Affiliations:** 1 UniSA STEM, University of South Australia, Adelaide, SA 5095, Australia; kit_man.chan@mymail.unisa.edu.au; 2 College of Medicine and Public Health, Flinders University, Bedford Park, SA 5042, Australia; krasimir.vasilev@flinders.edu.au; 3 Flinders Institute for Nanoscale Science & Technology, College of Science and Engineering, Flinders University, Bedford Park, SA 5042, Australia

**Keywords:** hexaminolevulinate, protoporphyrin IX, bladder cancer, fluorescence, PPOX, FECH, ABCG2, iron chelation

## Abstract

Seven different inhibitors of the heme metabolic pathway were applied in combination with HAL to study the formation of PpIX in bladder cancer HT1197 and normal fibroblast HFFF2 cells ex vivo, specifically with the aim to increase the fluorescence contrast between cancer and non-cancer cells. The mRNA expression of enzymes involved in the heme biosynthesis pathway were measured via PCR following incubation with the drugs in order to link the fluorescence levels and metabolic activity. The exogenous administration of HAL does lead to cancer-specific PpIX accumulation. However, the contrast between cancer and normal cells in suspension was not enhanced by the enzyme inhibitors and iron-chelating agents tested, nor did the mRNA expression necessarily correlate with the fluorescence intensity. The results indicate that a difference in the metabolic activity of cells in suspension may limit the applicability of exogenous enzyme inhibitor administration as a mean to improve the fluorescence-based detection of cancer cells shed in body fluids.

## 1. Introduction

Hexaminolevulinate (HAL) is a hexyl ester of 5-aminolevulinic acid (ALA), a precursor of the photosensitiser protoporphyrin IX (PpIX). Exogenous administration of 5-ALA or its derivatives increases the production of fluorescent endogenous PpIX via the heme biosynthesis pathway. Most importantly, PpIX has been reported to preferentially accumulate in cancer cells, when compared with normal cells, because of alteration in their heme synthesis and oncogenic metabolism [1]. When excited by blue light (~400 nm), cancer cells display red fluorescence because of the presence of intracellular PpIX. To date, ALA-induced PpIX fluorescence is clinically applied for the detection of various cancers and can enable early diagnosis [2,3,4,5]. HAL is more lipophilic than 5-ALA and induces higher PpIX fluorescence at a lower concentration compared with 5-ALA [6,7]. For these reasons, HAL has been approved by the FDA and the European Union for use in blue light cystoscopy for bladder cancer detection [8,9]. In previous works, the principles of HAL-induced PpIX fluorescence have been applied ex-vivo for the detection of bladder and prostate cancer cells shed in the urine of patients [10,11]. Albeit promising, clinical results could be strengthened by increasing the fluorescence contrast between malignant and healthy cells.

**Figure 1:**
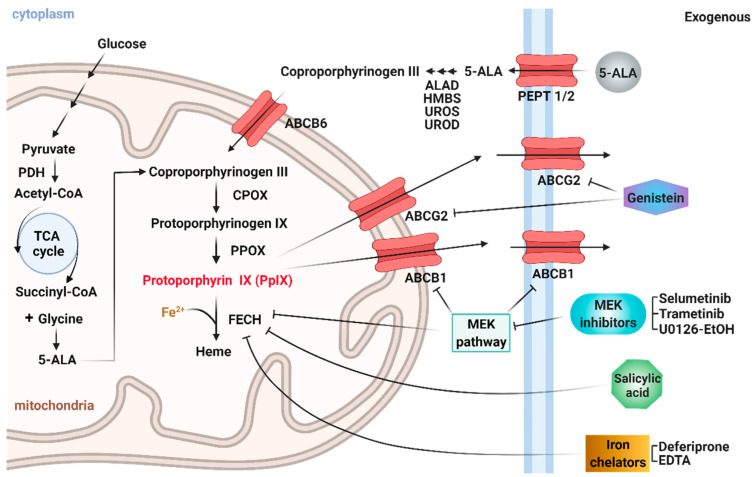
Schematic diagram illustrating the cellular mechanisms for increasing PpIX accumulation by different inhibitions. Created with BioRender.com.

Despite its established clinical application successes, the HAL-induced PpIX fluorescence intensity can be highly variable between cancer types, with some cancer cells exhibiting high PpIX fluorescence while others show very low or no PpIX fluorescence after ALA administration [12]. In some instances, the contrast can be insufficient to distinguish between cancer and normal cells. This heterogeneous PpIX accumulation can be caused by differences in cellular oxygen supply and heme metabolic activities. Indeed, PpIX is produced through a complex series of enzymatic reactions occurring between the mitochondrion and the cytoplasm (Figure 1). The whole enzymatic synthesis is controlled by glucose and heme concentrations through a feedback mechanism. Intracellular 5-ALA is produced from succinyl-CoA (which is generated in the glucose tricarboxylic acid TCA cycle) and glycine. Exogenously applied 5-ALA can induce significant intracellular levels of PpIX. First, exogenous 5-ALA is converted into coprotoporphyrinogen III by a succession of enzymatic reactions involving ALAD, HMBS, UROS and UROD. Next, coprotoporphyrinogen III is transported inside the mitochondria and oxidized by protoporphyrinogen III oxidase (PPOX) to finally generate PpIX. The porphyrin production steps are oxygen-dependent processes. In the following step, either ferrous iron (Fe^2+^) is chelated to PpIX by a rate-limiting enzyme ferrochelatase (FECH) to form heme in the mitochondria [13] or PpIX is transported to the cytoplasm through efflux transporters such as ATP binding cassette subfamily B member 1 (ABCB1) and ATP binding cassette subfamily G member 2 (ABCG2) [14]. Various inhibitors of these chelating and efflux steps of the metabolism have been shown to be effective in increasing PpIX accumulation in some cancer types [15], although results for bladder cancer have not been reported.

Results from our previous works indicated that the cell microenvironment plays an important role in the regulation of PpIX accumulation when suspended bladder cells are treated with HAL [16]. In this study, we hypothesize that reducing the PpIX efflux and FECH activity with inhibitors and iron chelators could enhance HAL-induced PpIX fluorescence within bladder cancer cells (Figure 1). The seven inhibitors investigated, and their expected mode of action, are summarized in Table 1. Genistein is an inhibitor of membrane transporter ABCG2, which is expected to reduce the PpIX efflux [17]. Salicylic acid (SA) binds to FECH and inhibits its enzymatic activity [18]. Deferiprone and EDTA are both iron chelators that bind to ferric irons and thereby hinder the conversion of PpIX into heme. Lastly, selumetinib, trametinib and U0126-EtOH are MEK inhibitors expected to limit the oncogenic MEK pathway. MEK inhibition has been shown to enhances PpIX accumulation in colon and some breast cancer cells by decreasing PpIX conversion to heme as well as limiting PpIX efflux through ABCB1 [15].

Here, we investigated the effect of these enzyme inhibitors and iron chelators on bladder cancer cell line (HT1197) and normal human fibroblasts (HFF2) in suspensions. Cell suspensions were used to mimic the ex vivo behaviour of cells found in body fluids, such as urine, after they have been shed by the tumour [22]. Bioassays performed on cell suspensions can inform on the usefulness of HAL-assisted photodynamic detection of cancer in non-invasive diagnostic approaches relying on urine samples (e.g., point of care devices) rather than solid tumour imaging (cystoscopy). The overall HAL-induced PpIX fluorescence was measured following incubation with HAL with and without the adjuvants. Different incubation times and temperature were tested. In addition, PCR was used to investigate the corresponding gene expression levels for PPOX, FECH and ABCG2 enzymes. 

## 2. Results

### 2.1. PpIX Fluorescence Measurement in Cells Distinctly Differ among Tested Drugs

HFFF2 and HT1197 cells were treated with HAL as previously described [23]. Briefly, the cells were suspended in a 50 µM HAL solution at a concentration of 2 × 10^5^ cells per mL. The cells were then treated with the inhibitors under investigation (EDTA, selumetinib, U0126, genistein, SA, deferiprone and trametinib), for a different amount of time and at different temperatures, as summarized in Table 2. The effect of the seven PpIX conversion and/or membrane transport inhibitors on the overall HAL-induced fluorescence of the cells was then quantified following established fluorescence microscopy procedures [16,23,24].

The PpIX fluorescence of the cancer cells treated with HAL is systematically and significantly higher than that of normal HFFF2 cells after 2 h incubation at 37 °C, without added inhibitors (** *p* ≤ 0.01, Figure S1). This is not always the case at 23 °C, the temperature at which ex-vivo analysis of urine samples would typically occur. 

**Figure 2:**
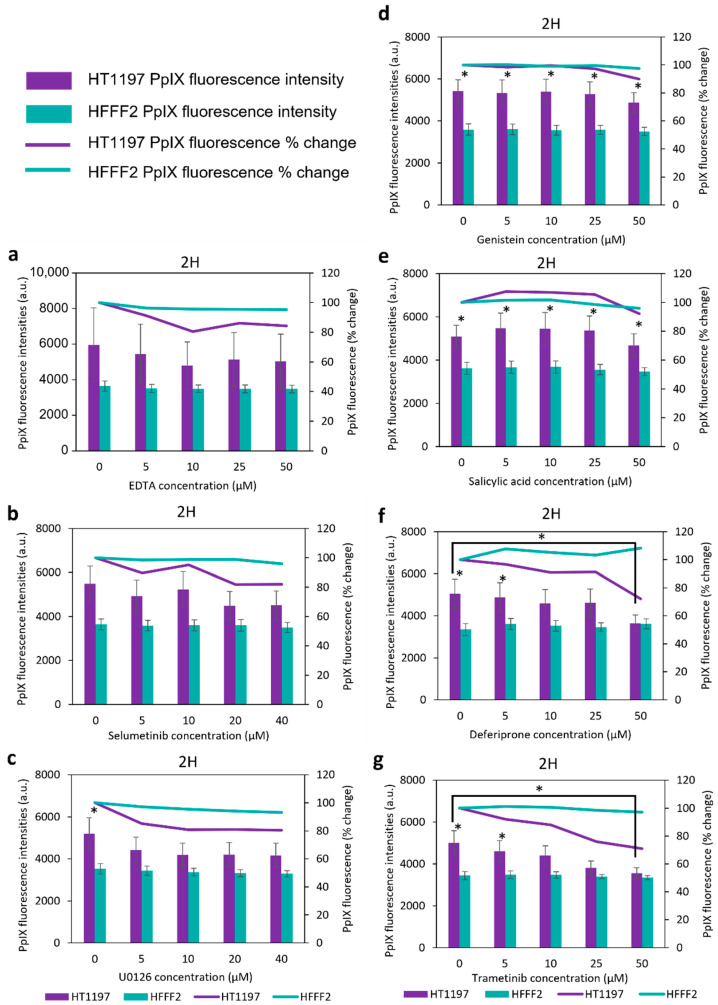
Mean PpIX fluorescence in bladder cancer (HT1197) and normal fibroblasts (HFFF2), treated with 50 µM of HAL and various concentrations of (**a**) EDTA; (**b**) selumetinib; (**c**) U0126; (**d**) genistein; (**e**) salicylic acid; (**f**) deferiprone; (**g**) trametinib for 2 h a 23 °C. Statistical analysis was performed using two-tailed Welch’s *t*-tests; * *p* ≤ 0.05.

The non-cancer HFFF2 cells did not experience any significant change in PpIX fluorescence intensity for any of the inhibitors and incubation conditions investigated (Figure S1, 2 h at 37 °C; Figure 2, 2 h at 23 °C; and Figure S2, 1 h at 23 °C).

In bladder cancer cells HT1197, the fluorescence tends to decrease following incubation with the inhibitors, though no significant changes were recorded after only 1 h incubation at 23 °C. (Figure S2). After 2 h, however, PpIX fluorescence in bladder cancer HT1197 cells decreased by up to 20% following incubations with 50 µM EDTA, selumetinib and U0126 (Figure 2a–c), and a significant 30% decrease occurred after incubation with 50µM deferiprone and trametinib (Figure 2f,g). In these cases, the decrease was so important that the absolute fluorescence of the cancer cells was no longer different from that of the normal cells. The greatest reduction in fluorescence intensity (35% reduction) was recorded when HT1197 cells were treated with 50 µM HAL and 25 µM deferiprone after 2 h incubation at 37 °C (Figure S1. These results indicate that the addition of inhibitors to cancer cells in suspensions has a detrimental effect on the overall HAL-induced fluorescence, especially at the highest concentration investigated. The only conditions leading to a measurable increase in fluorescence were incubations with 5 to 25 µM salicylic acid (SA) at 23 °C (5 to 7% promotion, Figure 2e).

These results, indicating that the inhibitors broadly failed to enhance PpIX accumulation, are counter-intuitive and prompted further investigation into the enzymatic activity levels within the cells.

### 2.2. PpIX Fluorescence Is Not Correlated with Expression of PPOX, FECH and ABCG2

**Figure 3:**
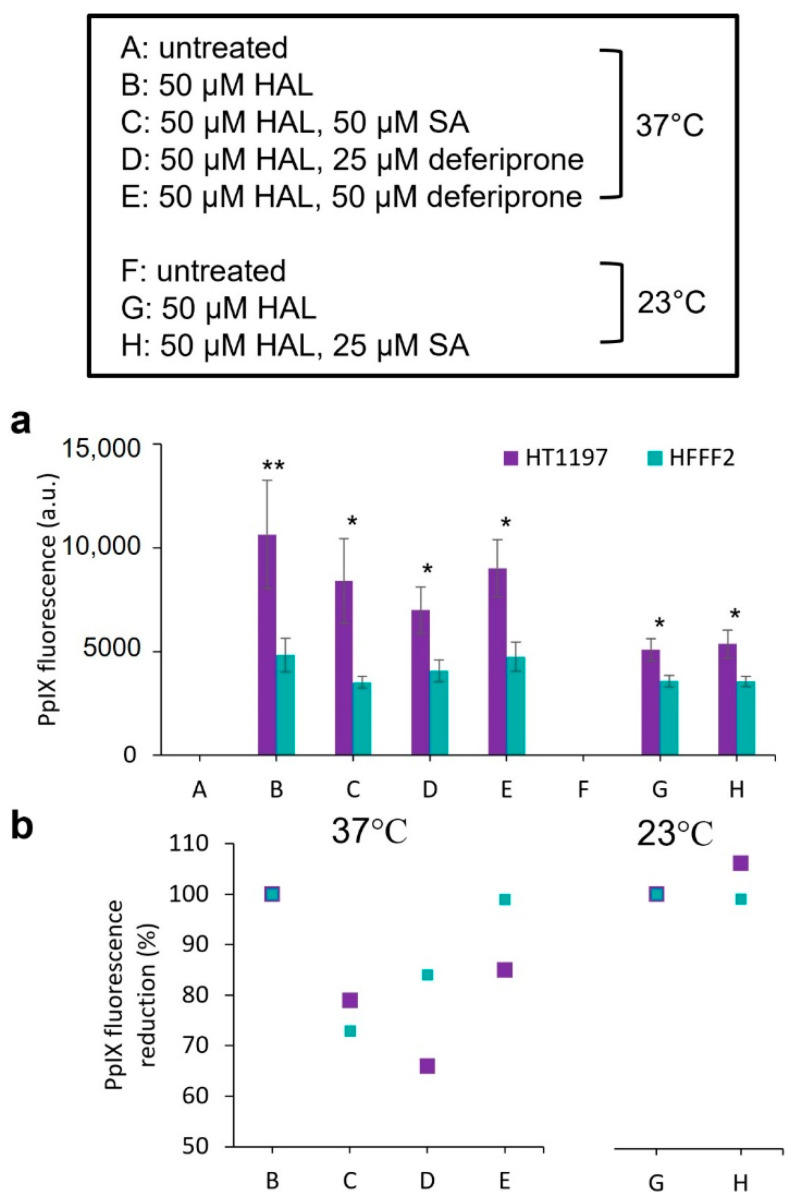
(**a**) Mean PpIX fluorescence in bladder cancer (HT1197) and normal fibroblasts (HFFF2), treated with 50 µM of HAL and drugs for 2 h in 37 °C and 23 °C; results also show the corresponding (**b**) percentages of PpIX fluorescence reduction in cells in conditions C, D and E compared to condition B (in 37 °C), and cells in conditions H compared to condition G (in 23 °C). Statistical analysis was performed using two-tailed Welch’s *t*-tests; * *p* ≤ 0.05; ** *p* ≤ 0.01.

Based on the results above, we selected the following conditions to test the effect of inhibitors on the cells enzymatic activities: salicylic acid (50 µM) and deferiprone (25 and 50 µM) at 37 °C, which induced a reduction in PpIX fluorescence; and salicylic acid 25 µM at 23 °C, which induced a PpIX fluorescence increase. The corresponding absolute PpIX fluorescence intensity and the percentage of PpIX fluorescence variation are summarized in Figure 3 together with results obtained for control cells that were only treated with HAL (Figure 3a,b).

**Figure 4:**
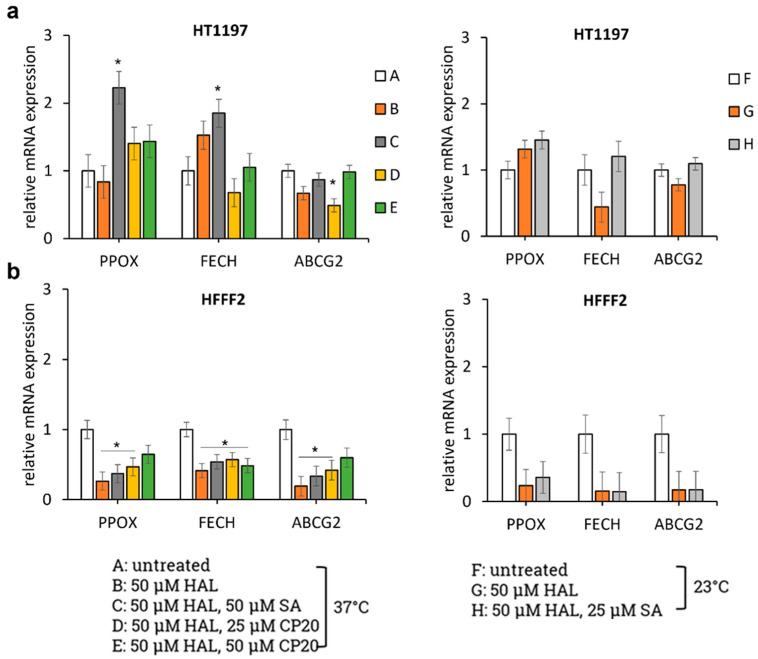
mRNA expression levels of the biosynthesis pathway-related genes PPOX, FECH and ABCG2 in (**a**) HT1197 and (**b**) HFFF2 cells. Statistical analysis was performed using two-tailed Welch’s *t*-tests; * *p* ≤ 0.05.

These results were used to investigate the effects of SA and deferiprone on the gene expression of both porphyrin biosynthesis enzymes (PPOX and FECH) and membrane transporter (ABCG2). The gene expression of PPOX, FECH and ABCG2 was examined via PCR after treatment with the drugs and were expressed relative to those of GAPDH (Figure 4). 

The drugs used in treatment C, D, E and H, namely, SA and deferiprone, are expected to downregulate FECH, the enzyme responsible for PpIX conversion to heme. For the non-cancer HFFF2 cells, a decrease in the level of all mRNA tested, FECH but also PPOX and ABCG2, was indeed observed following combination of all treatments.

Interestingly, treatment with both HAL and SA upregulated the expression of PPOX, an enzyme that was not targeted by these treatments. This increase was significant at 37 °C. Treatment with deferiprone at 37 °C also upregulated PPOX, albeit not significantly. PPOX contribute to the formation of PpIX and its upregulation is therefore expected to increase fluorescence, as seen in condition H.

For treated HT1197, the expression of ABCG2, which is responsible for PpIX efflux [25], was equivalent or downregulated (condition D) compared to untreated cells. This membrane transporter was not deliberately targeted by SA and deferiprone, and its downregulation with 25 µM deferiprone was not associated with an increase in PpIX fluorescence. 

The relative expression of FECH did not change significantly following treatment with deferiprone, and it was significantly higher in HT1197 cells treated with both HAL and SA at 37 °C (condition C). While this was not the expected action of SA, this result is consistent with the decrease in fluorescence observed following this treatment, as an increased FECH activity is expected to promote conversion of PpIX into heme.

Taken together, these results indicate that the PpIX accumulation induced by HAL with or without adjuvant drugs (SA or deferiprone) does not correlate directly with PPOX, FECH and ABCG2 regulation.

## 3. Discussion

Uncovering the molecular mechanism that drive cancer-specific PpIX fluorescence induced by exogenous drugs is essential for developing targeted strategies that can enhance PpIX accumulation, not only in vivo but also ex vivo. This is particularly relevant to urogenital cancers, or more precisely to their non-invasive diagnostic. As tumour cells from bladder and prostate cancer are readily shed in urine after detaching from the primary tumour site, their detection in urine sample could enable rapid screening and pain-less follow ups [26,27].

Here the correlation between adjuvant inhibitors and porphyrin biosynthesis pathway-related genes was investigated for model cell lines in suspension. Alike tumour cells shed in urine, cell lines in suspension have the unique characteristic of being in “survival mode”—finding themselves in less-than-optimum conditions in terms of temperature and media, and having most likely suffered some degree of membrane damage; the metabolic activity of suspended adherent cells is disturbed, including that of the heme biosynthesis pathway. To evaluate whether or not inhibitors known to affect PpIX accumulation in vivo prove efficient ex vivo, the fluorescence intensity of cells treated with HAL and adjuvant drugs were compared with those of untreated cells. The fluorescence intensity of non-cancerous HFFF2 cells did not change in the presence of the adjuvant. Only the cancer cells reacted to their addition, and this was the case not only for HT1197, but also other bladder cancer cell lines such as RT4 and HT1376 (Figure S3). These results show that the addition of drugs did impact PpIX accumulation, though it generally resulted in its decrease as a function of the drug concentration. A simple explanation for this could be that the drugs primarily compromised the cell membrane integrity, which would lead to effusion of PpIX outside the cells. Indeed, the iron chelator deferiprone readily enters cells and has been reported to impair membrane phospholipid turnover [28], while EDTA and salicylic acid are known to disrupt cell membrane permeability [29,30,31]. For cells in suspension, as opposed to monolayers or tumours, the cell membrane is exposed to the media from all sides, which may enhance the PpIX outflow arising from the lipid bilayer destabilization. In this case, PpIX effusion may well outweigh the concurrent effect the inhibitors have on enzymatic activity.

To test this hypothesis, enzymatic activities were tested via PCR for the conditions resulting in the most extreme change in overall fluorescence. mRNA expression levels of PPOX, FECH and ABCG2 were quantified. These three genes were chosen because PPOX is the enzyme generating PpIX from coprotoporphyrinogen III. FECH then catalyses PpIX into heme, and ABCG2 contributes to PpIX transmembrane efflux. Thus, PpIX accumulation is expected to correlate with PPOX upregulation, and FECH and ABCG2 downregulation. In vivo studies have shown that the downregulation of ABCG2 is a key molecular mechanism in PpIX accumulation in bladder cancer [25]. Yet, in the cell suspension studied here, the downregulation of ABCG2 measured for HT1197 in condition D (25 µM deferiprone) was not associated with an increase in fluorescence. The upregulation of PPOX following incubation with SA (condition C and H) was accompanied by the expected increase in PpIX fluorescence at 23 °C (condition H) but not at 37 °C, condition C. The decrease in fluorescence instead observed for condition C could be due to the concomitant upregulation of FECH. This result is in agreement with other studies that reported that FECH silencing led to PpIX accumulation [32]. Overall, however, the many discrepancies observed seem to indicate that the heme biosynthetic pathway, or its inhibition thereof, is not the main driving parameter when it comes to the absolute fluorescence of cells in suspension. Instead, a maximum fluorescence intensity is achieved owing to the availability of excess HAL, membrane porosity and optimum temperature.

The study presented here investigated the accumulation of fluorescent PpIX in bladder cancer cell line suspensions ex vivo, following the administration of exogenous HAL with or without iron chelator and enzyme inhibitors. HAL administration led to an increase in PpIX fluorescence intensity, which at 37 °C was significant compared to non-cancer HFFF2 cells. The concurrent administration of the adjuvants genistein, selumetinib, trametinib, U0126-EtOH, salicylic acid, EDTA and deferiprone, however, did not increase PpIX accumulation further. Instead, large concentration of drugs led to a decrease in fluorescence. PCR measurement suggest that the enzymes contributing to the heme metabolic pathway, such as FECH, respond differently to the addition of inhibitor when cells are in suspension. These results point to the limitation of HAL-induced fluorescence as a means to systematically discriminate healthy and cancer cells ex vivo, where the cell metabolic activity may be compromised.

## 4. Materials and Methods

### 4.1. Chemicals

Hexaminolevulinate (HAL) hydrochloride, Ethylenediaminetetraacetic acid (EDTA) and phosphate-buffered saline (PBS) tablets were purchased from Sigma-Aldrich, Castle Hill,, NSW, Australia. Genistein, selumetinib, trametinib, U0126-EtOH, salicylic acid and deferiprone were obtained from Selleck, Scoresby, VIC, Australia.

### 4.2. Cell Culture

Human bladder carcinoma HT1197 and human foetal foreskin normal fibroblast HFFF2 cell lines were supplied by the European Collection of Cell Cultures (ECACC; UK) and purchased from CellBank Australia, Westmead, NSW, Australia. HT1197 cells were cultured in MEME + 1% MEM nonessential amino acid solution from Sigma-Aldrich. HFFF2 cells were cultured in DMEM from Thermo Fisher Scientific, Brisbane, QLD, Australia. All culture growth media were supplemented with 10% foetal calf serum and 1% (*v*/*v*) penicillin/streptomycin. Cells were cultured at 37 °C in a humidified atmosphere containing 5% CO_2_.

### 4.3. HAL-Based and Drug Treatment

Trypsinized cell suspensions were centrifuged at 225× *g* for 5 min at room temperature. The supernatant was removed, and the cell pellet was resuspended in PBS at a density of 2 × 10^5^ cells per mL For the fluorescence intensity experiment, the cells were then incubated with 50 µM HAL and seven types of test solutions (0 to 40 or 50 µM of each tested drug) at 37 °C for 2 h or at 23 °C for the time of interest (1 to 2 h). For the enzymatic expression experiment, the cells were incubated for 2 h with 50 µM HAL and either 50 µM salicylic acid or 25 µM deferiprone at 37 °C or 25 µM salicylic acid at 23 °C. These specific conditions were selected from the first part experimentation. As untreated control, cells were treated without any HAL or drugs. 

### 4.4. PpIX Fluorescence Measurement

After the drug treatment, cells were pipetted into 96-well plates for PpIX fluorescence imaging through a custom made inverted fluorescent microscope. PpIX fluorescence was measured using the Image-Pro Premier software. Using built-in software thresholding settings, PpIX fluorescent cells that were distinguishable from the background pixels through the long pass filter (600 nm) were counted. The mean intensity of each object in triplicate (*n* = 3) were recorded in arbitrary units defined by the software. Control wells consisted of cells incubated with 50 µM HAL only without the tested drugs. Results were expressed as the mean ± standard deviation (SD) of the mean. Statistical analysis was performed using two-tailed Welch’s t-tests, to compare the differences in PpIX fluorescence produced by the different cell types at each condition. 

### 4.5. RNA Extraction, cDNA Synthesis and qRT-PCR

Total RNA was extracted from cell sediments with the RNeasy Plus Micro Kit (Qiagen, Clayton, VIC, Australia), and cDNA was synthesized from the extracted RNA using the QuantiTect Reverse Transcription Kit (Qiagen, Clayton, VIC, Australia) according to the manufacturer’s protocol and kept in −20 °C until use. qRT-PCR was performed using the LightCycler 480 Instrument II (Roche Diagnostics, North Ryde, NSW, Australia), and the following TaqMan Gene Expression Assays were used: PPOX (Hs00970229_g1), FECH (Hs01555261_m1), ABCG2 (Hs01053790_m1) and GAPDH (Hs02786624_g1). The qRT-PCR was run at 50 °C for 2 min, followed by 95 °C for 10 min and 50 cycles of amplification at 95 °C for 15 s and 60 °C for 1 min. Each sample was performed in triplicate. The 2^−ΔΔCT^ method was used to normalize the mRNA and the relative mRNA expression level was compared to an untreated control (expressed as 1).

## Figures and Tables

**Table 1:** Summary of the adjuvant drug tested types, targets, pathway and respective actions (down arrows indicates down-regulation), as reported in the literature [15,17,18,19,20,21].

Drug	Type	Target	Pathway	Expected Action
Selumetinib	Enzyme inhibitor	MEK1/2	Ras/MEK MAPK/ERK	ABCB1 🠗 FECH 🠗
U0126-EtOH				
Trametinib				
Salicylic acid		COX	Nuclear factor-ĸB	FECH 🠗
Genistein		ABCG2	Heme	ABCG2 🠗
EDTA	Iron chelators	Iron chelators		FECH 🠗
Deferiprone				

**Table 2:** List of the different parameter investigated: inhibitors, concentrations, cell types, incubation time and temperatures.

Inhibitors	EDTA, Selumetinib, U0126, Genistein, SA, Deferiprone and Trametinib
Concentrations	5–50 µM
Cell types	HT1197, HFFF2
Incubation time	1 h, 2 h
Incubation temperature	23 °C, 37 °C

## Data Availability

The data presented in this study are available in supplementary material.

## Supplementary Materials

The following supporting information can be downloaded at: https://www.mdpi.com/article/10.3390/ijms23147631/s1.
