# Supplementary material for: Effects of Supplemental Drugs on Hexaminolevulinate (HAL)-Induced PpIX Fluorescence in Bladder Cancer Cell Suspensions
Source: Int J Mol Sci. 2022 Jul 10;23(14):7631. doi: 10.3390/ijms23147631 (PMC9323055; doi:10.3390/ijms23147631)
Supplement: Supplementary file 1 [file ijms-23-07631-s001.zip › ijms-1793584-supplementary.pdf]

International Journal of *Molecular Sciences*

Article

Effects of Supplemental Drugs on Hexaminolevulinate (HAL)-Induced PpIX Fluorescence in Bladder Cancer Cell Suspensions

Kit Man Chan [1], Krasimir Vasilev [2] and Melanie MacGregor [3,*]

1 UniSA STEM, University of South Australia, Adelaide, SA 5095, Australia; kit_man.chan@mymail.unisa.edu.au
2 College of Medicine and Public Health, Flinders University, Bedford Park, SA 5042, Australia; krasimir.vasilev@flinders.edu.au
3 Flinders Institute for Nanoscale Science & Technology, College of Science and Engineering, Flinders University, Bedford Park, SA 5042, Australia
* Correspondence: melanie.macgregor@flinders.edu.au; Tel.: +61-8-8201-2574

International Journal of
Molecular Sciences

MDPI

Supplementary Materials

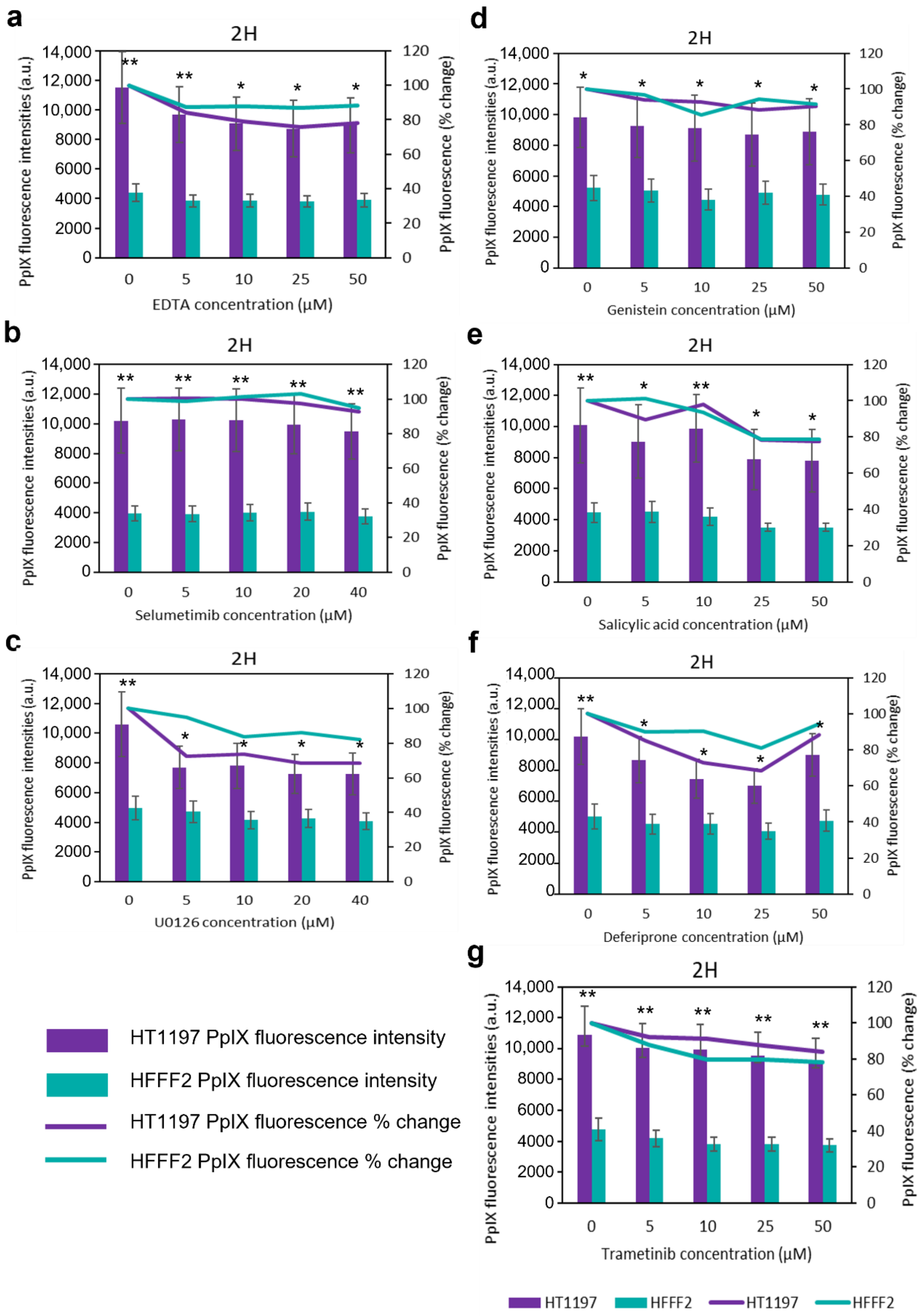

Figure S1. Mean PpIX fluorescence in bladder cancer (HT1197) and normal fibroblasts (HFFF2), treated with 50 µM of HAL and drugs for 2 hours in 37°C. Statistical analysis was performed using two-tailed Welch's t-tests; * p ≤ 0.05; ** p ≤ 0.01.

International Journal of
Molecular Sciences

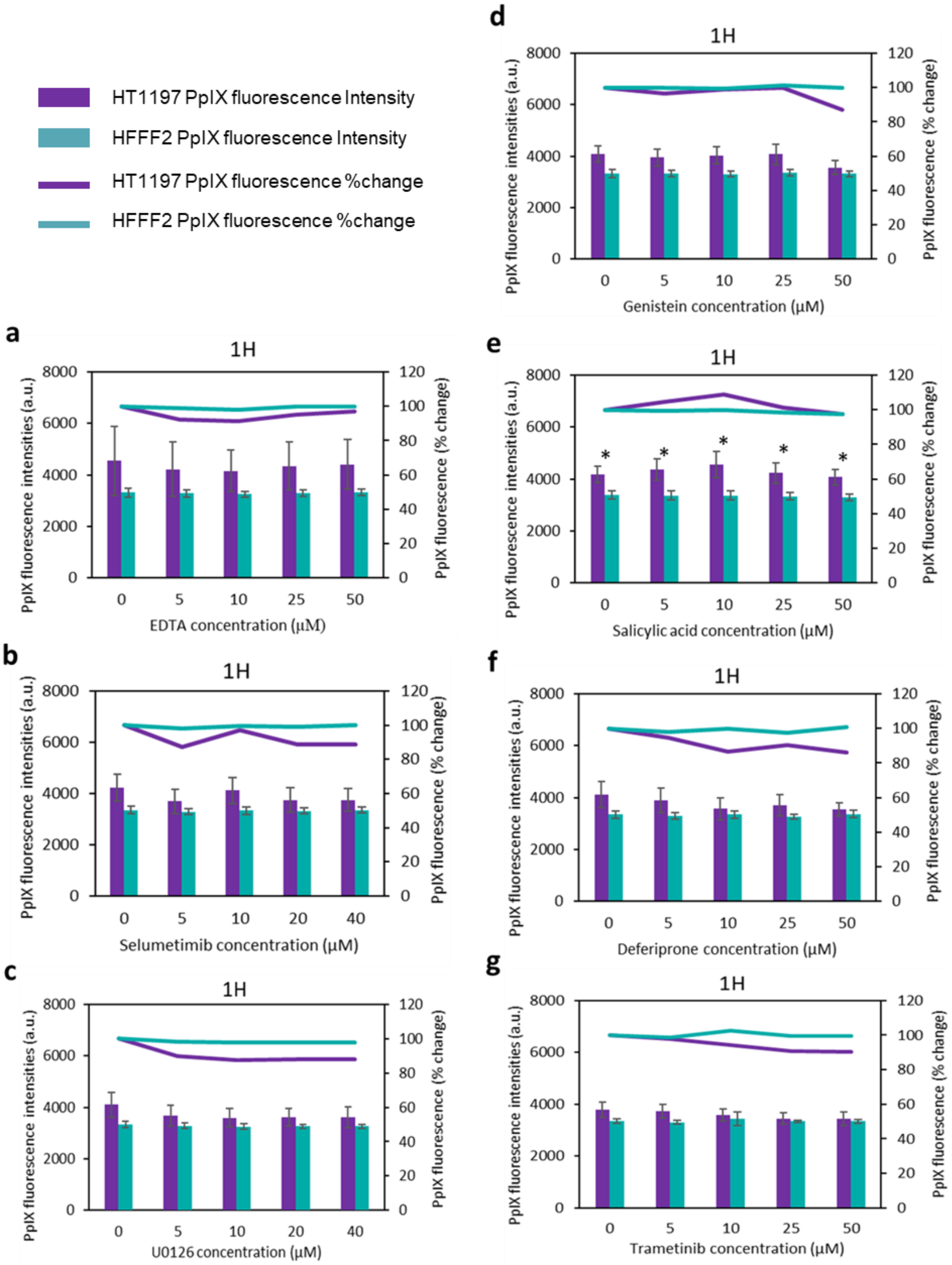

Figure S2. Mean PpIX fluorescence in bladder cancer (HT1197) and normal fibroblasts (HFFF2), treated with 50 μM of HAL and drugs for 1 hour in 23°C. Statistical analysis was performed using two-tailed Welch's t-tests; * p ≤ 0.05.

International Journal of *Molecular Sciences*

MDPI

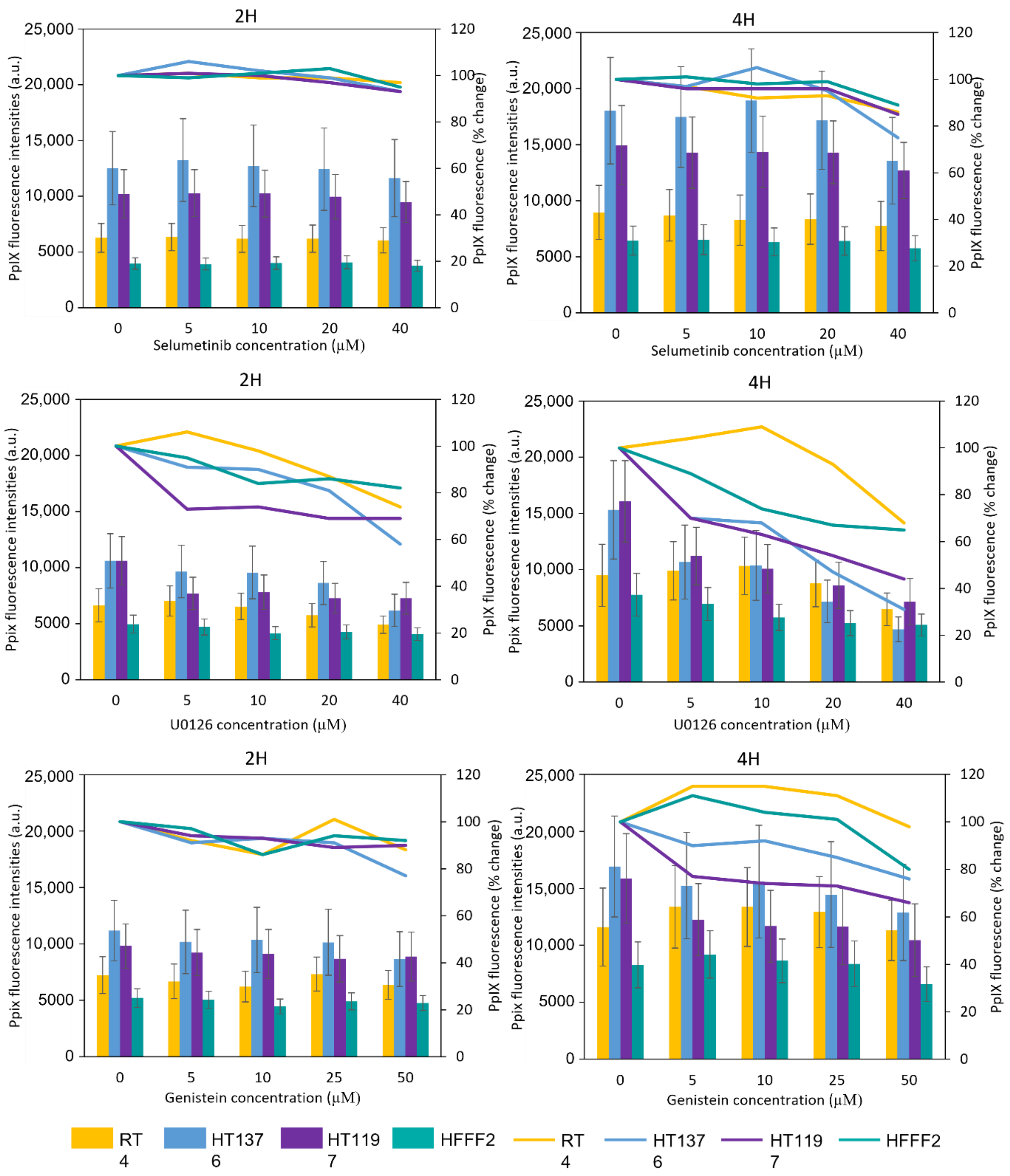

Figure S3. Mean PpIX fluorescence in bladder cancer (RT4, HT1376, HT1197) and normal fibroblasts (HFFF2), treated with 50 µM of HAL and drugs for 2 and 4 hours in 37°C.
